# New Insight Into Pathogenicity and Secondary Metabolism of the Plant Pathogen *Penicillium expansum* Through Deletion of the Epigenetic Reader SntB

**DOI:** 10.3389/fmicb.2020.00610

**Published:** 2020-04-09

**Authors:** Joanna Tannous, Omer Barda, Dianiris Luciano-Rosario, Dov B. Prusky, Edward Sionov, Nancy P. Keller

**Affiliations:** ^1^Department of Medical Microbiology and Immunology, University of Wisconsin – Madison, Madison, WI, United States; ^2^Institute of Postharvest and Food Sciences, The Volcani Center, Agricultural Research Organization, Rishon LeZion, Israel; ^3^Department of Plant Pathology, University of Wisconsin – Madison, Madison, WI, United States; ^4^College of Food Science and Engineering, Gansu Agricultural University, Lanzhou, China; ^5^Food Research Institute, University of Wisconsin – Madison, Madison, WI, United States; ^6^Department of Bacteriology, University of Wisconsin – Madison, Madison, WI, United States

**Keywords:** *Penicillium expansum*, epigenetic regulation, SntB, epigenetic reader, virulence, secondary metabolism, patulin, citrinin

## Abstract

*Penicillium expansum* is one of the most harmful post-harvest pathogens of pomaceous fruits and the causal agent of blue rot disease. During infection, *P. expansum* produces the toxic secondary metabolites patulin and citrinin that can impact virulence and, further, render the fruit inedible. Several studies have shown that epigenetic machinery controls synthesis of secondary metabolites in fungi. In this regard, the epigenetic reader, SntB, has been reported to govern the production of multiple toxins in *Aspergillus* species, and impact virulence of plant pathogenic fungi. Here we show that deletion of *sntB* in *P. expansum* results in several phenotypic changes in the fungus including stunted vegetative growth, reduced conidiation, but enhanced germination rates as well as decreased virulence on Golden Delicious apples. In addition, a decrease in both patulin and citrinin biosynthesis *in vitro* and patulin in apples, was observed. SntB positively regulates expression of three global regulators of virulence and secondary metabolism (LaeA, CreA, and PacC) which may explain in part some of the phenotypic and virulence defects of the PeΔ*sntB* strain. Lastly, results from this study revealed that the controlled environmental factors (low temperatures and high CO_2_ levels) to which *P. expansum* is commonly exposed during fruit storage, resulted in a significant reduction of *sntB* expression and consequent patulin and citrinin reduction. These data identify the epigenetic reader SntB as critical factor regulated in post-harvest pathogens under storage conditions and a potential target to control fungal colonization and decaying of stored fruit.

## Introduction

The ubiquitous fungus *Penicillium expansum* is the dominant post-harvest pathogen among fruits and vegetables, mainly pome fruits (Tannous et al., 2017a). Because of its ability to grow at low temperatures, *P. expansum* has also been associated with widespread spoilage of fruits during storage (Altunatmaz et al., 2012; Tannous et al., 2015). Besides the aesthetic aspect of its presence, contamination by *P. expansum* poses a health hazard due to the production of toxic secondary metabolites (mycotoxins) in contaminated fruit. Among the mycotoxins produced by *P. expansum*, the polyketide patulin is the most notable contaminant, given its long-established toxicity and prevalence during fruit infection (Puel et al., 2010; Tannous et al., 2017a). Citrinin is another polyketide mycotoxin reported to often co-occur with patulin on apples (Martins et al., 2002). This mycotoxin has been shown to be genotoxic and nephrotoxic (Flajs and Peraica, 2009). Due to the agricultural losses and serious health risks associated with the occurrence of these mycotoxins, a deeper understanding of what triggers their production is needed.

A few years ago, the complete genome sequence of *P. expansum* was unveiled by two research groups (Ballester et al., 2015; Li et al., 2015). Both papers provide new insights into secondary metabolism biosynthetic gene clusters, mainly those responsible for patulin and citrinin biosynthesis. Today, the biosynthetic pathways of patulin and citrinin synthesis are largely known (Artigot et al., 2009; Snini et al., 2014; Tannous et al., 2014, 2017b; He and Cox, 2016; Li et al., 2019). Recent studies were focused on the regulation of these two mycotoxins and other *P. expansum* secondary metabolites that could impact virulence and/or health hazards related to this pathogen. In this regard, the widely studied global regulator of secondary metabolism in fungi, LaeA, a member of the transcriptional Velvet Complex (Bayram et al., 2008), was shown to positively regulate patulin production (Kumar et al., 2016). Deletion of *laeA* in two *P. expansum* strains led to a significant decrease in patulin biosynthesis due to down-regulation of the expression of the patulin biosynthesis genes (Kumar et al., 2016). In contrast, citrinin biosynthesis was not subjected to *laeA* regulation in *P. expansum* (Kumar et al., 2016). Both patulin and citrinin synthesis were reduced as a result of deletion of *veA*, a second component of the Velvet Complex (El Hajj Assaf et al., 2018). Patulin was found to also be positively regulated by PacC, the pH regulatory transcription factor (Barad et al., 2014; Chen et al., 2018). Lastly, CreA, the carbon catabolite repressor has been reported to act as a positive regulator of both patulin and citrinin production (Tannous et al., 2018). In addition to their impact on secondary metabolism and some physiological traits, the loss of all these regulatory proteins resulted in attenuated virulence of *P. expansum* on apples (Kumar et al., 2016; El Hajj Assaf et al., 2018; Tannous et al., 2018).

In recent years, epigenetic and consequent histone post-translational modifications have been shown to play an important role in secondary metabolite production in fungi (Pfannenstiel and Keller, 2019), including regulation of several *Aspergillus* and *Fusarium* mycotoxins such as aflatoxin, fumonisin and trichothecenes (Visentin et al., 2012; Liu et al., 2015; Yang et al., 2016; Pfannenstiel et al., 2018). Most studies investigated the role of histone modifying enzymes, such as methylases or histone deacetylases in modulating secondary metabolite synthesis in fungi. However, more recently an additional histone modifying protein, the epigenetic reader SntB, was identified in *Aspergillus* species to regulate not only fungal development and secondary metabolism (Pfannenstiel et al., 2017) but also virulence in the aflatoxigenic fungus *A. flavus* (Pfannenstiel et al., 2018). SntB orthologs, called Snt2, had previously been described in the plant pathogens *Fusarium oxysporum* (Denisov et al., 2011) and *Magnaporthe oryzae* (He et al., 2018). These studies did not address the impact on secondary metabolism but found that the loss of *snt2* resulted in reduced virulence of both pathogens on their respective hosts. Additionally, both studies reported defects in autophagy-dependent cell death pathways in the *snt2* mutants.

To our knowledge, epigenetic control of mycotoxin production and virulence in *P. expansum* has not been addressed. Our work presents evidence that SntB regulates *P. expansum* development, patulin and citrinin production, and virulence on apples. SntB is a positive regulator of *laeA, creA*, and *pacC* expression, which may explain some of its impact on fungal biology. Moreover, this is the first report of epigenetic response(s) of a post-harvest pathogen to environmental stressors. Data from our study revealed a deregulation of *sntB* under low temperatures and high CO_2_ levels, two conditions widely applied during fruit storage to limit *P. expansum* growth. These results allowed us to propose epigenetic responses as a potential modulator of virulence of storage pathogens.

## Materials and Methods

### Strains and Growth Conditions

*Penicillium expansum* strain Pe-21 (also called Pe-d1) from Israel, was used to generate the Δ*ku70* knock-out strain that was used as the parental control strain for all subsequent experiments. Derivative strains from *P. expansum* Pe-21 are listed in Supplementary Table S1. Strains were cultivated in glucose minimal medium (GMM) at 25°C for 5 days to collect fresh spores. Conidia were collected and adjusted using a hemocytometer to the indicated concentrations. Screening of fungal transformants was done on sorbitol minimal medium (SMM) agar supplemented with the appropriate antibiotic (hygromycin or phleomycin at the respective concentrations of 100 and 50 μg/ml). For physiological experiments, GMM agar and broth were used. To screen for secondary metabolite production *in vitro*, strains were cultivated on four different agar media chosen to represent a broad range of nutrient source, which are: Potato Dextrose Agar (PDA), Yeast Extract Sucrose Agar (YES), Czapek Yeast Extract Agar (CYA) and GMM, at 25°C for 10 days. Each medium was prepared as shown in Supplementary Table S2.

### Plasmid and Strain Construction and Confirmation

Competent cells of the yeast strain BJ5464 (*MATalpha*, *ura3*-*52*, *trp1*, *leu2*-*Δ1*, *his3*-*Δ200*, *pep4:HIS3*, *prb1*-*Δ1.6R*, *can1*, *GAL*) were used to facilitate appropriate plasmid construct assembly. The plasmids used in this study are listed in Supplementary Table S3.

#### Construction of the ku70 Gene Deletion Cassette

In our recent work, a Δ*ku70* strain of *P. expansum* Pe-21 was generated using hygromycin as a selectable marker (Tannous et al., 2018). Here a new *P. expansum* Δ*ku70* was created to allow recycling of the hygromycin resistance gene that can be re-used as a selectable marker in future transformations. The new *P. expansum* Δ*ku70*, hygromycin sensitive (Hyg^S^) strain, termed TJT14.1, was constructed by employing the β-Rec/six site-specific recombination system described by Hartmann et al. (2010). The hygromycin recyclable marker used to construct the Δ*ku70* strain is based on site-specific recombination that allows repetitive gene targeting. The elements of the self-excising β-rec/*six* blaster cassette are shown in Supplementary Figure S1A. The Δ*ku70* knockout cassette was constructed using yeast recombination cloning (Wiemann et al., 2018). The three fragments (5′flank Ku70- hygromycin resistance cassette and 3′flank Ku70) were assembled into the pYHC-WA-pyrG plasmid. The plasmid was linearized by PCR using primers YS_F and YS_R (Supplementary Table S4A). After yeast recombinational cloning, the plasmid pJT1 was created and recovered from yeast DNA by transforming into *E. coli.* The nested primers Pe_KOKu70_NestedF and Pe_KOKu70_NestedR were used to amplify the entire deletion cassette from plasmid pJT1 using the Long Template Expand PCR System (Roche, Indianapolis, IN, United States). PCR reactions were performed according to the manufacturer’s instructions, and the construct was eluted with a QIAquick PCR Purification Kit (Qiagen, Hilden, Germany). This construct was used to transform protoplasts of the parental strain Pe-21 (Barad et al., 2016b).

#### Construction of sntB Gene Deletion and Complementation Cassettes

The predicted sequence for the *P. expansum sntB* ortholog was obtained from GenBank (XM_016748327) by conducting a BLAST search with the *A. flavus sntB* gene sequence (AFLA_029990) against the *P. expansum* genome scaffolds. The generated Hyg^S^ Δ*ku70* strain, TJT14.1, was used to delete the *sntB* gene using the phleomycin resistance gene *ble* as a selectable marker (Silar, 1995). The resultant deletion strain was called TJT15.1. The schematic representation of the *sntB* gene replacement with the *ble* gene is depicted in Supplementary Figure S2A. The three amplified fragments (5′flank *sntB*, *ble* gene, and 3′flank *sntB*) were assembled using a double joint PCR protocol described by Lim et al. (2012). To confirm that the phenotype exhibited by the Δ*sntB* strain is caused by the deletion of this specific gene, TJT15.1 was complemented with a wild-type copy of the *sntB* gene using hygromycin as selectable marker to create TJT17.1. *Not*I and *Bsrg*I restriction sites introduced respectively at the predicted promoter and terminator of the *sntB* gene, using primers Pe_sntBcomp_F and Pe_sntBcomp_R, were used to subclone *sntB* downstream of the *hph* gene into the pJT1 plasmid (Supplementary Figure S3A). The ligation of the digested insert into the recipient plasmid was performed using the T4 DNA ligase from New England Biolabs, according to the manufacturer’s instructions. The ligation reaction was later transformed into *E. coli* DH5alpha competent cells following the manufacturer’s directions (Thermo Fisher Scientific). Five bacterial colonies were picked and screened for successful ligations by conducting a diagnostic restriction digest with the same enzymes used for the cloning. The correct plasmids were then grown in 50 ml LB supplemented with ampicillin (100 μg/ml), and the plasmid DNA was isolated using Quantum Prep^®^Plasmid Midiprep Kit (Biorad) according to the manufacturers’ instructions. Ten micrograms of plasmid DNA was used for the transformation of the Δ*sntB* strain. The plasmid was linearized using *swa*I prior to fungal transformation.

#### Protoplast Transformation

To generate the different deletion and complement strains, the fungal transformation was performed following the polyethylene glycol-mediated protoplast transformation protocol described previously by Tannous et al. (2018). All transformants were screened by PCR and further subjected to southern blot to confirm the single insertion of the deletion cassette using [α32P] dCTP (PerkinElmer, United States) to label the DNA probes, following manufacturer’s instructions. In order to recycle the *hph* resistance cassette and gain back the sensitivity to hygromycin B in the Δ*ku70* strain generated in this study, deletion strains were grown on minimal medium amended with 2% xylose to activate the ß-recombinase placed under the xylose-inducible promoter. The confirmation of the hygromycin sensitivity was done on GMM hygromycin plates and by PCR targeting the *hph* gene. Primers used for constructing the deletion cassettes, molecular cloning and mutants’ confirmation are listed in Supplementary Table S4A.

### Physiological Analysis

Both vegetative growth and conidial production were assessed on GMM agar. To measure the radial mycelial growth, agar plates were point-inoculated with 10^6^ spores of each strain and incubated at 25°C. Radial growth was monitored by diameter measurements in the four cardinal directions. Measurements were recorded on alternate days for a 6 day period.

To quantify total conidia produced by the various strains, GMM top agar (0.7% (w/v) agar) containing 10^6^ spores was overlaid on agar plates of the same media (20 ml) and incubated at 25°C in the dark. To accurately count conidia, 2 cm plugs from each plate were homogenized in 3 ml of 0.01% Tween 20 water, diluted and enumerated with a hemocytometer. Conidial production was quantified starting by the second day post-inoculation using three replicate plates per strain.

Germination assays were performed in sterile 12-well culture plates. The spore concentration of all strains was adjusted to 10^5^ spores/ml in GMM broth, and one milliliter of each spore suspension was distributed into 3 replicate wells. Time-course microscopy was carried out over 24 h at 25°C using a Nikon Ti inverted microscope. Germination rate was monitored regularly by capturing images of each well hourly, beginning 4 h post-incubation. A number of spore germlings was counted for each strain and recorded. The percent of germinated spores was plotted against time, and the germination rates were determined.

### Evans Blue Staining

Liquid cultures of *P. expansum* strains were grown in GMM supplemented with Yeast Extract (YE). The 25 ml cultures were inoculated using 10^8^ spores and incubated for 14 h at 25°C under 250 rpm. After incubation, mycelia were harvested in 50 ml tubes and washed twice with Dulbecco’s Phosphate-Buffered Saline (DPBS) lacking calcium and magnesium (Life Technologies, Carlsbad, CA, United States). For the negative control, mycelia were incubated at 100°C for 45 min before proceeding with the protocol. Mycelia were later incubated at 37°C for 10 min in a 0.1% Evans Blue (Sigma Aldrich, St Louis, MO, United States) solution. After incubation, three wash steps were performed using DPBS again. Samples were mounted using Ibidi mounting Medium (Ibidi, Fitchburg, WI, United States).

### Imaging and Analysis

Fluorescence microscopy was performed using Axio Imager A10 Microscope EC Plan-NEOFLUAR 40x/1.3 Oil DIC/∞/0.17 objective and a series 120 X-Cite R light source (EXFO) (Olympus Corporation, Shinjuku, TYO, Japan). Micrographs were acquired using the DIC (Differential Interphase Contrast) and TRITC (Tetramethylrhodamine) channels with uniform exposure settings for all samples: 6 ms for the DIC channel and 40 ms for the TRITC channel. The mean gray value area per hyphae of 10 micrographs per strain was calculated using Fiji (Schindelin et al., 2012).

### RNA Isolation and qRT-PCR Analyses

Cultures intended for RNA isolation were grown on sterile 0.45 μm nitrocellulose membrane circles (Whatman, Kent, United Kingdom) placed on top of solid YES medium (20 g bacto yeast extract, 150 g sucrose, 15 g bacto agar per liter). A 10^6^ fungal spores/ml solution (100 μl) was inoculated onto 55 mm petri dishes containing 10 ml of media. To test the impact of abiotic stressors, the plates were incubated at 28°C for 48 h in the dark and then transferred to the appropriate stress conditions for additional 48 h. For comparison between the WT, Δ*sntB* and complement strains, the plates were grown at 28°C for 4 days. After incubation, total RNAs were extracted from 100 mg of lyophilized mycelia using the Hybrid-R RNA isolation kit (GeneAll, Seoul, South Korea) according to the manufacturer protocol. The DNase and reverse-transcription reactions were performed on 1 μg of total RNA with the Maxima First-Strand cDNA Synthesis Kit (Thermo Fisher Scientific, Waltham, MA, United States) according to the manufacturer protocol. The cDNA samples were diluted 1:20 (v/v) with ultrapure water. Real time quantitative PCR was performed with the StepOnePlus system (Applied Biosystems, Waltham, MA, United States). PCR amplifications were performed with 3 μl of cDNA template in 10 μl of a reaction mixture containing 7 μl mix from the Fast SYBR green Master Mix (Applied Biosystems, Waltham, MA, United States) and 200 nM primers. PCRs were carried out with the following cycling program: 20 s at 95°C, followed by 40 cycles of 95°C for 3 s and 60°C for 20 s. The samples were normalized using β*-tubulin* gene (PEXP_025370) as endogenous control and the relative expression levels were measured using the 2^(–ΔΔCt)^ analysis method (Livak and Schmittgen, 2001). Primers used for qRT-PCR analyses are listed in Supplementary Table S4B. Results were analyzed with StepOne software v2.3.

### Mycotoxin Analysis

Agar plugs with fungal mycelia from gene expression experiments were used to evaluate mycotoxin production. For patulin analysis, 1 g agar was added to 2 ml of HPLC grade ethyl acetate (Bio-Lab, Jerusalem, Israel) and crushed to homogeneity. Patulin was extracted by shaking for 30 min at 150 RPM on an orbital shaking platform. The supernatant was transferred to a clean glass tube and was evaporated to dryness under a stream of gaseous nitrogen at 50°C. The residues were re-dissolved in 1 ml of the mobile phase (0.02 M ammonium acetate:acetonitrile 9:1), filtered through a 0.22 μm PTFE syringe filter (Agela Technologies, Tianjin, China) and kept at −20°C prior to HPLC analysis. For citrinin analysis, 1 g agar plug with fungal mycelia was added to 1 ml of methanol and crushed. The mycotoxin was extracted by shaking for 30 min at 150 RPM and centrifuged for 10 min at 6000 × g. The supernatant was filtered through a 0.22 μm PTFE syringe filter and kept at −20°C prior to HPLC analysis. The citrinin mobile phase consisted of acidified water (adjusted to pH 2.5 with acetic acid) and acetonitrile (50:50). Both mycotoxins were quantitatively analyzed by injection of 20 μl into a reverse phase UHPLC system (Waters ACQUITY Arc, FTN-R, Milford, MA, United States) using a Kinetex 3.5 μm XB-C18 (150 × 4.6 mm) column (Phenomenex, Torrance, CA, United States). The column temperature was maintained at 30°C and the flow rate was 1 ml/min. The patulin peak was detected with a photodiode array (PDA) detector at 280 nm; citrinin was detected with a fluorescence detector (331 nm excitation, 500 nm emission). Both mycotoxins were quantified by comparing with a calibration curve of the standard mycotoxins (Fermentek, Jerusalem, Israel).

### Virulence Assessment of *P. expansum* Strains

*SntB* deletion and complement mutants confirmed by Southern blot analysis were tested on apples cv. Golden Delicious, obtained from a local orchard, to evaluate the role of *sntB* in the virulence process of *P. expansum*. As described previously by Tannous et al. (2018), apples were surface sterilized with 2% sodium hypochlorite solution and thoroughly rinsed with sterile distilled water. A single uniform 5 mm deep wound was made at the equator of each apple (put on its side) using sterile toothpicks. For each strain, 10 μl of a spore suspension at a concentration of 10^8^ spores/ml were deposited into the wound. Apples were incubated at 25°C in the dark for 11 days. The diameter of the rotten spots were recorded every other day. Three technical replicates were performed for each strain, and the entire experiment was repeated twice for confirmation of the observed results. At the end of the incubation period, patulin was extracted from apples and quantified by HPLC as described earlier by Tannous et al. (2018). Moreover, to eliminate the effect of variability between apples and to compare the fruit colonization, the three strains (control, Δ*sntB*, and *sntB*C) were inoculated twice on the same apple. Six technical replicates were performed and the whole experiment was repeated three times for confirmation of the observed results. Representative images were taken 5 days post-inoculation.

### Statistical Analyses

For all experiments, values are stated as the mean ± standard error of the mean (SEM) of three independent replicates unless otherwise indicated. The statistical analysis of the data was performed using the one-way ANOVA. If one-way ANOVA reports a *p* value of < 0.05 further analyses were performed using Tukey’s single-step multiple comparison test to determine significant difference between the strains. Analyses were done using R Studio (R Studio Team, 2015, Boston, MA, United States) and GraphPad Prism software (GraphPad, San Diego, CA, United States).

## Results

### Multi-Species Comparison of SntB Protein Sequences

The deduced amino acid sequence of SntB in *P. expansum* (XP_016595169) showed a high degree of identity with orthologous sequences from species of *Aspergillus* (ranging from 60 to 64%), whereas a lower percentage of identity was scored with the orthologous sequences from *M. oryzae* (around 46%) and *F. oxysporum* (around 42%). The sequence identity with the Snt2 of *Saccharomyces cerevisiae* was even lower (around 32%), and therefore it was excluded from the multisequence alignment. Alignment of deduced amino acid sequences of SntB revealed the presence of five highly conserved domains (boxes A–E) that are considered to be essential for the activity of this protein (Supplementary Figure S4). The first conserved domain (box A) consists of the Bromo adjacent homology (BAH) domain that may be involved in protein-protein interaction specialized in gene silencing (Callebaut et al., 1999). Box B carries the first plant homeodomain (PHD) finger which is a C_4_HC_3_ zinc-finger-like motif thought to be involved in epigenetics and chromatin-mediated transcriptional regulation (Aasland et al., 1995; Fair et al., 2001). The third domain (box C) contains the SANT (SWI3, ADA2, N-CoR, and TFIIIB) DNA-binding domains, belonging to a various set of proteins that share a common 3 alpha-helix bundle. Recent studies suggested that SANT domains might be a histone-tail-binding module (Boyer et al., 2004). In boxes D and E, are found two other plant homeodomain (PHD) fingers.

### Strains Construction and Validation by Southern Blotting Analysis

To identify the Hyg^S^ Δ*ku70:hph* transformants, fifty colonies obtained using the pJT1 vector were found resistant to hygromycin. Monosporic transformants were further checked by direct specific PCR analysis targeting the *ku70* gene using the primer set Pe_Ku70orf_F and Pe_Ku70orf_R. Amplification of the ORF was not observed in twenty three transformants, which were then analyzed by southern blotting. Only five out of twenty three transformants showed a single insertion of the cassette at the *ku70* locus. To recycle the *hph* resistance cassette and gain back the sensitivity to hygromycin B, these five transformants were grown on minimal medium amended with 2% xylose. Four of these transformants were not able to grow on plates supplemented with hygromycin B after this treatment. The absence of *hph* gene was further confirmed by PCR using the primer set hph_F and hph_R (data not shown). A single Δ*ku70*, Hyg^S^ strain labeled TJT14.1 was used for the following experiments as a control strain. Southern blot confirmation for the selected Δ*ku70* is shown in Supplementary Figure S1B.

To determine the effects of *sntB* on growth, conidiation, and other cellular processes, *sntB* deletion (*Pe*Δ*sntB*) and complementation strains were generated. For *sntB* deletion, ten single transformants were initially selected on SMM agar supplemented with phleomycin (50 μg/ml). The phleomycin resistant strains were later confirmed by PCR for the absence of the ORF amplification (data not shown) and subjected to a final confirmation by southern blot analysis using probes specific for flanking regions of the *sntB* gene (Supplementary Figure S2B). Two DNA fragments of 1216 and 1189 kb corresponding respectively to the 5′ and 3′flanking regions of *sntB* were used to generate the radioactive probes utilized in this hybridization. Genomic DNA from both the control and *Pe*Δ*sntB* strains were digested separately with *Eco*RI and *Ban*II. The presence of 5.4 and 2.5 kb bands in the Southern blot analysis confirmed the *sntB* deletion, while 4.2 and 1.6 kb bands corresponded to the control (Supplementary Figure S2B). One of the correct *Pe*Δ*sntB* deletion strains was used for the following experiments and was called TJT15.1.

With respect to the *sntB* complement strain, diagnostic PCR was performed to confirm the integration of the wild type allele in the Δ*sntB* strain using the same set of primers that amplify the *sntB* ORF (data not shown). The positive strains were later subjected to southern blot analysis using the *sntB* gene as a probe (Supplementary Figure S3B). Genomic DNA from both *sntB* deletion and complement strains were digested with *Not*I and *Bsrg*I. As expected, the Δ*sntB* strain did not show any band, whereas the *sntB*c strain revealed the expected band of 6.3 kb (Supplementary Figure S3B). One of the correct *sntBc* strain was used for the following experiments and was labeled TJT17.1.

### *P. expansum* Δ*sntB* Is Aberrant in Spore Production, Germination, and Growth Morphology

**FIGURE 1:**
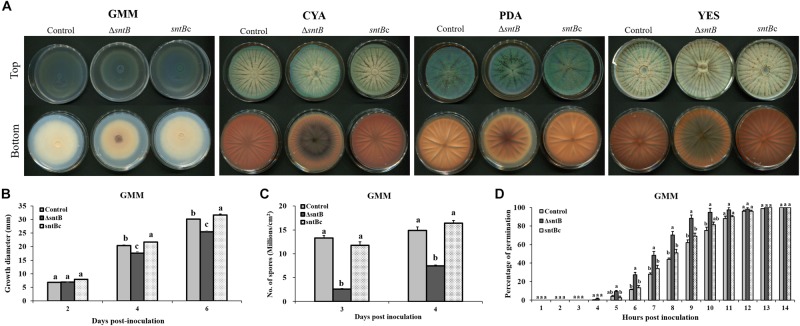
Physiological analysis of the control, *sntB* deletion and complement strains of *P. expansum* Pe-21. **(A)** Growth phenotype of *P. expansum* strains monitored on four different media (GMM, Glucose Minimal Medium; CYA, Czapek Yeast Extract Agar; PDA, Potato Dextrose Agar; YES, Yeast Extract Sucrose Agar) for 72 h at 25°C. **(B)** Radial growth, **(C)** conidia production were monitored on GMM agar, and **(D)** germination rate of *P. expansum* strains was measured on GMM broth. One-way ANOVA differences were considered significant when *p* < 0.05. Different letters above the columns indicate statistically significant differences between the strains as determined using Tukey’s single-step multiple comparison test.

The impact of *sntB* deletion on *P. expansum* physiology was evaluated by virtue of four different determinants: pigmentation, radial growth, spore production and germination rate. Visual observation of growth plates showed distinct pigmentation patterns correlating with *sntB* loss on all media examined after 72 h of growth at 25°C (Figure 1A). Radial growth data collected on GMM agar after 6 days of culture at 25°C revealed a significant reduction of growth diameter when *sntB* is deleted in *P. expansum* (Figure 1B). While the Δ*sntB* strain (TJT15.1) reached an approximate growth diameter of 25 mm, the control strain (TJT14.1) grew to an area of 30 mm, 6 days post-inoculation. However, the *sntB* complement strain (TJT17.1) was 1 mm greater in growth diameter compared to the control strain at the end of the incubation period (Figure 1B). A similar pattern was observed on the three other tested media (Supplementary Figures S5A–C). Sporulation data gathered on the second and third days post-inoculation on GMM agar showed decreased spore production by the Δ*sntB* mutant compared to both the control and the *sntB* complement strains (Figure 1C). It remains unclear if the reduced spore count in the Δ*sntB* strain is due to growth impairment. However, the *Pe*Δ*sntB* mutant spores showed a faster germination rate compared to the control strains, starting 4 h post-inoculation. All strains reached equivalent germination rates 11 h post-inoculation (Figure 1D).

### SntB Does Not Affect Cell Viability *in vitro*

Other studies have reported SntB homologs to be involved in autophagy mediated programed cell death (Denisov et al., 2011; He et al., 2018). To investigate if *P. expansum* SntB plays a role in cell death pathways, we first assessed *in vitro* cell viability of WT, Δ*sntB*, and *sntB* complement strains using Evans Blue Dye (Supplementary Figure S6A). Dead cells are permeable to this dye. Moreover, this staining fluoresces upon excitation with green light providing the opportunity to have quantitative data. After quantification and comparison of fluorescence using the average mean gray area for 10 micrographs per strain, we found no significant difference in cell viability between the assessed samples (Supplementary Figure S6B).

### SntB Modulates Mycotoxin Biosynthesis

**FIGURE 2:**
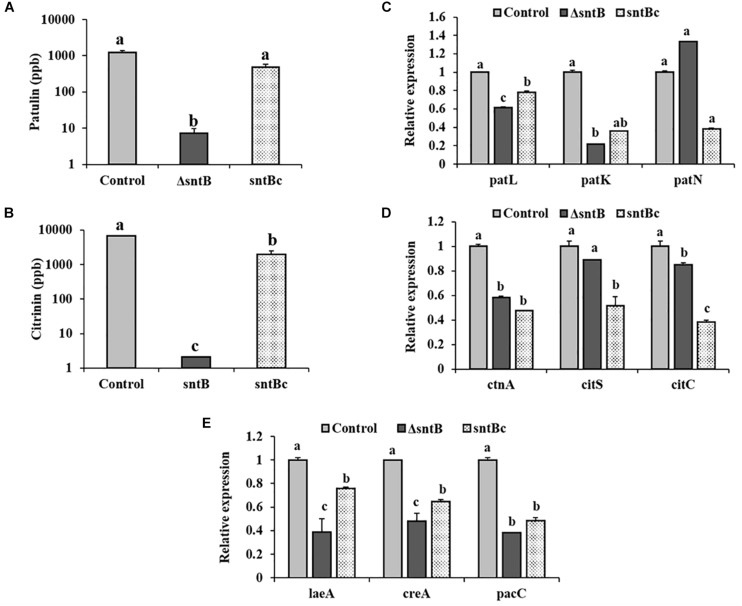
SntB-associated regulation of patulin and citrinin biosynthesis. The three *Penicillium* strains (control, Δ*sntB*, and *sntB*c) were grown on solidified YES media at 28°C. Patulin **(A)** and citrinin **(B)** levels, relative expressions of patulin **(C)** and citrinin cluster genes **(D)**, and global transcription factors **(E)** were evaluated at 4 days post-inoculation. Error bars represent the standard error of the mean (SEM) across three technical replicates. One-way ANOVA differences were considered significant when *p* < 0.05. Different letters above the columns indicate statistically significant differences between the strains as determined using Tukey’s single-step multiple comparison test.

Loss of *sntB* resulted in significant reduction of patulin and citrinin levels in the mutant strain compared to the control strain when grown in YES media (Figures 2A,B). To determine if the changes in patulin and citrinin levels were correlated with transcript levels, the expression levels of selected biosynthetic genes in both patulin and citrinin gene clusters were analyzed and compared between the control, *sntB* deletion and complement strains. Comparison of gene expression between the control and Δ*sntB* mutant revealed that deletion of *sntB* resulted in a reduced expression level of the specific transcription factor of the patulin gene cluster, *patL*, and the polyketide synthase (PKS) gene, *patK* (Figure 2C). Similarly, *sntB* deletion resulted in a significant reduction of the expression level of the citrinin gene cluster transcription factor, *ctnA*, and *citC*, which encodes an oxidoreductase in citrinin biosynthetic pathway (Figure 2D). Production of both mycotoxins was recovered by the complement strain, although not to the level of the control strain, which was in agreement with gene expression analysis (Figures 2A–D). These results were not totally unexpected considering that previous publications have reported similar outcomes, where the complement strains partially restored the parental phenotype (Carvalho et al., 2010; Kwon et al., 2011; Pfannenstiel et al., 2017).

Moreover, we also asked if loss of *sntB* influenced expression of regulatory genes known to regulate both metabolites. Expression of *creA* encoding the transcription factor regulating carbon catabolite repression (Tannous et al., 2018), *pacC* encoding the pH regulatory transcription factor (Chen et al., 2018) and *laeA* encoding a methyltransferase involved in global regulation of fungal secondary metabolism (Kumar et al., 2016), were markedly downregulated in the knockout strain (>2-fold, *p* < 0.001) (Figure 2E). Overall, the complement strain expressed similar levels of the global regulatory genes compared to the control strain (Figure 2E).

### SntB Deletion Mutant Exhibits Reduced Virulence and Patulin Production on Apples

**FIGURE 3:**
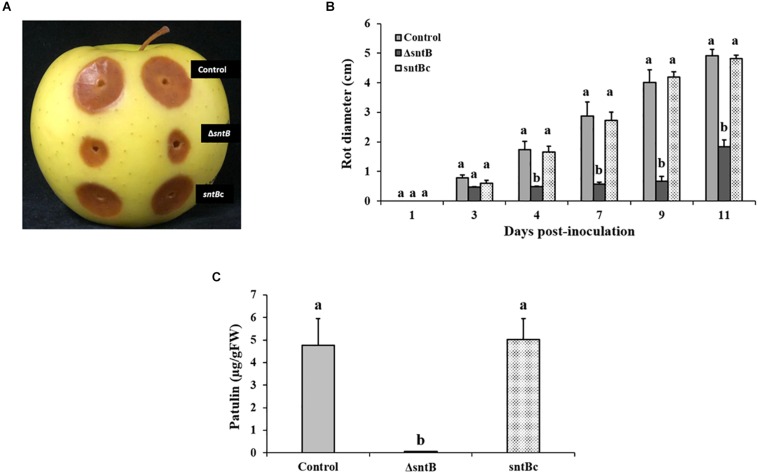
SntB is required for *P. expansum* pathogenicity and patulin production on apples. **(A)** Apples were inoculated with 10^6^ spores and incubated at 25°C in the dark for 5 days. **(B)** Histogram showing the rot diameter on apples inoculated with the three *Penicillium* strains (control, Δ*sntB*, *sntB*c). **(C)** Histogram displaying patulin production on apples analyzed by HPLC. Each strain was inoculated with 3 reps. One-way ANOVA differences were considered significant when *p* < 0.05. Different letters above the columns indicate statistically significant differences between the strains as determined using Tukey’s single-step multiple comparison test.

Next, we analyzed the ability of the *Pe*Δ*sntB* mutant to infect apple cv. Golden Delicious. Apple fruits were inoculated with a conidial suspension of Δ*sntB, sntB*c, and the *P. expansum* control strains. Lesion development was monitored every 2 days, and the diameter was measured. As shown in Figures 3A,B, *sntB* disruption resulted in a significant reduction of decay development as the lesion diameter caused by Δ*sntB* was about 37% smaller than that of the control and *sntB* complement strains on day 11 post-inoculation. Similar to the results described *in vitro*, the production of patulin by the Δ*sntB* mutant was significantly reduced compared to the control and the *sntB* complement strain on apples (Figure 3C). As described in our previous work (Tannous et al., 2018), citrinin was not detectable on apples.

### *sntB* and Subsequent Patulin and Citrinin Synthesis Is Regulated by Storage Environments

**FIGURE 4:**
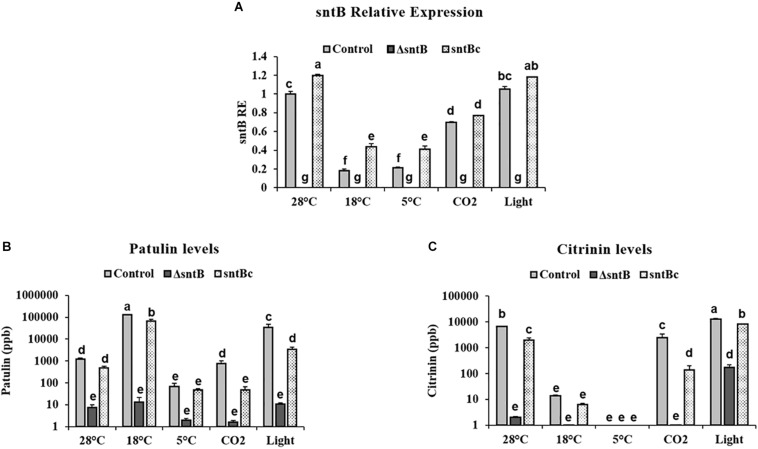
Effect of abiotic factors on *sntB* expression and mycotoxin accumulation. The three *Penicillium* strains (control, Δ*sntB*, and *sntB*c) were grown on YES agar media at 28°C for 48h in the dark and then subjected to different treatment conditions for additional 48 h. Control – 28°C incubation in the dark; 18–18°C incubation in the dark; 5–5°C incubation in the dark; light – 28°C incubation under continuous white fluorescent lighting; CO_2_ – 28°C incubation at 20% CO_2_ in the dark. **(A)** Total RNA was extracted and the relative expression of *sntB* was evaluated. **(B)** Patulin and **(C)** citrinin were extracted and subjected to HPLC analysis. Each strain was inoculated with 3 reps. One-way ANOVA differences were considered significant when *p* < 0.05. Different letters above the columns indicate statistically significant differences between the strains as determined using Tukey’s single-step multiple comparison test.

**FIGURE 5:**
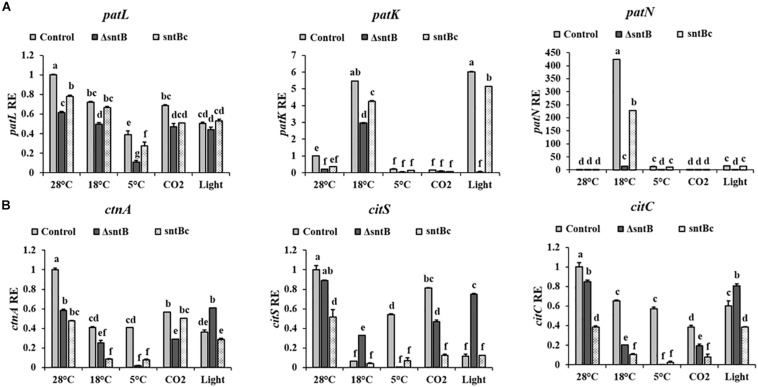
Effect of abiotic factors on relative expression of mycotoxin cluster genes. The three *Penicillium* strains (control, Δ*sntB*, and *sntB*c) were grown on YES agar media at 28°C for 48 h in the dark and then subjected to different treatment conditions as mentioned in Figure 4. RNA was extracted and the relative expression of patulin cluster genes **(A)**, and citrinin cluster genes **(B)** was evaluated. Error bars represent the standard error of the mean (SEM) across three technical replicates. One-way ANOVA differences were considered significant when *p* < 0.05. Different letters above the columns indicate statistically significant differences between the strains as determined using Tukey’s single-step multiple comparison test.

To gain an understanding on how SntB itself may be regulated and possibly affect subsequent patulin and citrinin synthesis in conditions associated with fruit storage, we assessed the impact of temperature, light and CO_2_ levels on gene expression and mycotoxin synthesis. qRT-PCR analyses showed a significant downregulation of *sntB* when *P. expansum* was exposed to high CO_2_ levels and low temperatures (18 and 5°C) (Figure 4A). Metabolite analysis showed that patulin and citrinin levels were reduced after incubation at 5°C and high CO_2_, while their synthesis was significantly upregulated under continuous white light (Figures 4B,C). Interestingly, incubation of the control strain at 18°C resulted in a dramatic increase in patulin accumulation of more than 130 ppm compared to 1.2 ppm at 28°C (control condition). In contrast, citrinin production by the control strain was markedly inhibited at 18°C to a concentration of 0.014 ppm compared to 6.8 ppm under control condition (Figures 4B,C). The *sntB* deletion strain produced significantly lower amounts of both mycotoxins compared to the control strain, while their accumulation was recovered by the complement strain, under all tested conditions (Figures 4B,C). A similar pattern was observed for the relative expression of the two key genes in the patulin biosynthetic pathway, *patK* and *patN* (Figure 5A). However, the transcript level of the patulin specific transcription factor (*patL*) was significantly decreased at 18°C and under white light, in contrary to the aforementioned increase of the mycotoxin level under the same conditions. The expression levels of the citrinin transcription factor (*ctnA*) and another two keys genes involved in citrinin biosynthesis (*citS* and *citC*) correlated with the mycotoxin production under all tested conditions except white light, under which a downregulation of the tested genes was observed (Figure 5B).

## Discussion

Due to its cytotoxic, genotoxic and teratogenic properties, its wide presence in fruits and its virulence-associated function (Snini et al., 2015), patulin synthesis by the fruit pathogen *P. expansum* has been the topic of research of many studies resulting in an excellent understanding of the biosynthetic pathway of this mycotoxin (Li et al., 2019). Emphasis has also been recently focused on citrinin, another nephrotoxic mycotoxin of *P. expansum*, reported to often co-occur with patulin (Gimeno, 2005). The biosynthetic steps leading to citrinin and the gene cluster responsible for its production are currently known (He and Cox, 2016; Schmidt-Heydt et al., 2019). Yet, the regulatory pathways underlying the production of both mycotoxins are still not completely resolved, and concurrent research is dedicated to expanding current knowledge.

This paper reports on the identification of a new protein in *P. expansum* that adds to the short list of proteins recognized to play a role in the virulence of this pathogen (Barad et al., 2016a; Kumar et al., 2016; Chen et al., 2018; El Hajj Assaf et al., 2018; Tannous et al., 2018; Li et al., 2019). This current study is the first to explore a novel response of the post-harvest pathogen *P. expansum* to epigenetic perturbations through the deletion of the epigenetic reader SntB. In addition to its impact on the ability of the fungus to cause infection and proliferate within apple tissue, SntB also regulates secondary metabolism in *P. expansum* with negative effects on patulin and citrinin production *in vitro* (Figures 2A,B).

*Penicillium expansum sntB* encodes a protein with several histone interacting domains: a BAH domain, a SANT domain and three plant homeodomain (PHD) fingers. SntB is homologous to Snt2 in *Saccharomyces cerevisiae* and *Schizosaccharomyces pombe* (Roguev et al., 2004; Baker et al., 2013). In the latter, Snt2 was shown to be a member of a protein complex called Lid2C that includes the proteins Lid2, Ash2, Sdc1, and Jmj3 (Roguev et al., 2004). A different complex was found in *S. cerevisiae*, and this includes the subunits Ecm5 and Rpd3 (Shevchenko et al., 2008). Originally reported to be simply involved in the regulation of chromatin remodeling (Roguev et al., 2004), subsequent research has shown that the protein reader SntB is a putative virulence factor and a major regulator of fungal secondary metabolism (Denisov et al., 2011; Baker et al., 2013; He et al., 2018; Pfannenstiel et al., 2018). Loss of *sntB* in the mycotoxigenic seed pathogen *A. flavus* resulted in global misregulation of secondary metabolism, where some metabolites were up- and some down-regulated such as aflatoxin (Pfannenstiel et al., 2018; Greco et al., 2019). We see a similar global misregulation in the *P. expansum*Δ*sntB* mutant.

The effect of SntB on various secondary metabolites may in part lay in its regulation of transcriptional complexes/transcription factors (Figure 2E). LaeA, a member of the Velvet Complex (VC) linking secondary metabolism to development (Bayram et al., 2008), regulates the expression of 12 putative backbone genes in *P. expansum* including patulin genes (Kumar et al., 2016). Along the same line, the carbon catabolite repressor, CreA, was proposed to post-transcriptionally regulate patulin and citrinin biosynthesis (Tannous et al., 2018). Similarly, deletion of *sntB* resulted in decreased biosynthesis of both mycotoxins (Figures 2A,B). Lastly, the reduced production of mycotoxins can also be potentially linked to the deregulation of the gene encoding the regulatory protein PacC (Figure 2E). Previous works have established that the pH-responsive zinc finger transcription factor PacC is essential for patulin biosynthesis in *P. expansum.* Barad et al. (2016a) provided the first evidence for the pivotal role of PacC in regulating patulin production in *P. expansum*. This paper showed that ammonia secretion by *P. expansum* during fruit colonization resulted in the activation of PacC and the subsequent increase of patulin accumulation (Barad et al., 2016a). These findings have later been confirmed by Chen et al. (2018) that showed a loss of patulin production by the Δ*pacC* mutant of *P. expansum* at pH values above 6.0 due to the significant down-regulation of all the genes in this cluster.

Whilst the deletion of *sntB* in *P. expansum* was not lethal, a large set of physiological characteristics were disturbed. This included decreased radial growth on all tested media, reduced conidial formation and accelerated spore germination (Figure 1). To the best of our knowledge, this is the first report to show the physiological effects of the epigenetic reader SntB in *Penicillium*. However, those findings were not unexpected as similar outcomes for *sntB* loss were observed in other ascomycetes. In *A. flavus*, the deletion of *sntB* has led to various morphological phenotypes, such as reduced radial growth and reduced sclerotia formation (Pfannenstiel et al., 2018). Along the same line, *snt2* deletion mutants of *Fusarium oxysporum* and *Neurospora crassa*, displayed morphological aberrations, including a drop in conidia formation and biomass accumulation, delayed vegetative growth and recurrent hyphal septation (Denisov et al., 2011; Kronholm et al., 2016). Although there have been several studies involved with SntB in ascomycetes, further data are needed to establish a congruent storyline about the impact of SntB on fungal development. Several previous reports described the role of epigenetics in the regulation of phenotypic alterations in filamentous fungi. Epigenetic mechanisms, e.g., histone modifications and RNA interference pathway, were found to be involved in phenotypic plasticity (i.e., phenotypic changes in the colony morphology) of *N. crassa* across an array of controlled environments including temperature, pH, osmotic stress and sugar content (Kronholm et al., 2016). Comparable results were previously gathered in the dimorphic yeast *Aureobasidium pullulans*, where phenotypic switches from yeast to mycelial form are likely to be an epigenetic phenomenon (Slepecky and Starmer, 2009).

Apart from their impact on fungal morphology and secondary metabolism, a number of studies highlighted the crucial role of epigenetic modifications in the success of fungal plant pathogens. Virulence assays performed on apples showed that the deletion of the *sntB* gene had a profound effect on lesion development during infection as lesion diameter caused by Δ*sntB* was smaller than that of the control strain (Figure 3). Complementation of the deletion strain with the wild type copy of *sntB* restored the parental virulence phenotype (Figure 3). Our results were in agreement with previous reports on three other plant pathogens, *A. flavus* (Pfannenstiel et al., 2018), *M. oryzae* (He et al., 2018), and *F. oxysporum* (Denisov et al., 2011), where the deletion and/or disruption of *sntB* reduced virulence on the respective plant hosts.

How SntB regulates *P. expansum* virulence is still unknown. The studies of both *M. oryzae* and *F. oxysporum* suggested that decrease in virulence could be mediated by the autophagic programmed cell death. However, our results did not support a role for SntB in PCD in *P. expansum* (Supplementary Figure S6). One possible explanation for these results is the difference in protein architecture of *P. expansum* SntB compared to other reported species. In *M. oryzae*, MoSnt2 contains an ELM2 domain that *P. expansum* SntB lacks (He et al., 2018). In a similar manner, *F. oxysporum* f. sp. *melonis* Snt2 contains a GATA-Zn domain that is not present in *P. expansum* SntB (Denisov et al., 2011). The reported functional difference may also be explained by the distinctive lifestyles of plant pathogenic fungi. In *M. oryzae*, autophagy mediated cell death and MoSnt2 are required for appressoria formation, an essential penetration structure present in *M. oryzae* but not in *P. expansum* (Veneault-Fourrey et al., 2006; He et al., 2018).

*Penicillium expansum sntB* was found to be regulated by different environmental factors, such as temperature and CO_2_ levels, that are usually used to modulate the physiology of stored fruits (Hulme, 1971). Moreover our data revealed that the down regulation of *sntB* observed at low temperatures and high CO_2_ levels (Figure 4A) is accompanied by a significant decrease of patulin and citrinin production (Figures 4B,C). However, we found a negative correlation between patulin accumulation and *patL* expression in the WT under certain environmental conditions (incubation at 18°C and under light; Figure 5A). In addition to transcription factor of the patulin gene cluster (PatL), other regulatory proteins known to impact patulin biosynthesis in *P. expansum*, including transcription factors LaeA, CreA, and PacC associated with production of secondary metabolites and broad responses to carbon availability and pH, respectively (Kumar et al., 2016, 2018; Chen et al., 2018; Tannous et al., 2018). At the same time, patulin accumulation under all tested conditions is positively correlated with the transcript level of one of the key genes in the patulin biosynthetic pathway, *patK* (PKS). Those results are of great importance as they point out that the same environmental factors that regulate the host physiological response also regulate the expression of *P. expansum sntB* and mycotoxin production simultaneously. Therefore, the epigenetic reader SntB opens up new avenues of study in order to thoroughly understand *P. expansum* biology and virulence. This will allow better regulation of fungal decaying of stored fruit as well as mycotoxin contamination by *P. expansum* during fruit storage.

## Conclusion

An increasing body of evidence points toward epigenetic mechanisms being responsible for a wide array of biological phenomena, including morphological development, virulence and secondary metabolism. This work represents the first step in the exploration of epigenetic regulation of development and pathogenesis in the post-harvest plant pathogen *P. expansum.* The present data shows that SntB is necessary for the biosynthesis of several secondary metabolites, including patulin and citrinin, and is required for full virulence on apples. *sntB* expression responds to environmental factors modulated during host storage and thus may provide clues to incorporate epigenetic control strategies against this storage disease.

## Data Availability Statement

All datasets generated for this study are included in the article/Supplementary Material.

## Author Contributions

NK, DP, ES, OB, and JT conceived the presented idea and designed the study. JT, OB, and DL-R carried out the experiments, collected, analyzed the data and designed the figures. JT wrote the manuscript with support from OB and DL-R on specific sections. NK, DP, and ES edited the manuscript. All authors discussed the results, commented on the manuscript, conceived, and planned the experiments.

## Conflict of Interest

The authors declare that the research was conducted in the absence of any commercial or financial relationships that could be construed as a potential conflict of interest.
