# Supplementary material for: New Insight Into Pathogenicity and Secondary Metabolism of the Plant Pathogen Penicillium expansum Through Deletion of the Epigenetic Reader SntB
Source: Front Microbiol. 2020 Apr 9;11:610. doi: 10.3389/fmicb.2020.00610 (PMC7160234; doi:10.3389/fmicb.2020.00610)
Supplement: Supplementary file 1 [file Data_Sheet_1.PDF]

Table S1. *Penicillium expansum* strains used in this study

**Strain ID**	**Genotype**	**Reference**
Pe-21	Wild Type	Ballester et al. 2015
TJT14.1	Δ*ku70::hph*[a], Hyg^{S*}	This study
TJT15.1	Δ*ku70::hph*, Δ*sntB::ble*[b], Hyg^{S}, $Phleo^{R*}$	This study
TJT17.1	Δ*ku70::hph*, Δ*sntB::ble, sntB::hph*, Hyg^{R*}, $Phleo^{R}$	This study

[a] *hph*: *E.coli* hygromycin B phosphotransferase gene.

[b] *ble*: *Streptoalloteichus hindustanus (Sh) ble* gene, encoding a 14kDa phleomycin binding protein.

* Hyg^{S}, Hyg^{R} and $Phleo^{R}$ refer to hygromycin-sensitive, hygromycin-resistant and phelomycin-resistant strains, respectively.
