# Supplementary material for: New Insight Into Pathogenicity and Secondary Metabolism of the Plant Pathogen Penicillium expansum Through Deletion of the Epigenetic Reader SntB
Source: Front Microbiol. 2020 Apr 9;11:610. doi: 10.3389/fmicb.2020.00610 (PMC7160234; doi:10.3389/fmicb.2020.00610)
Supplement: Supplementary file 2 [file Data_Sheet_2.PDF]

Table S2. Composition of culture media used in this study.

Media	Ingredients (per Liter)
**GMM**	50ml 20x Nitrate salts [a]<br>1ml Trace elements [b]<br>10g Dextrose<br>16g Agar
**SMM**	50ml 20x Nitrate salts<br>1ml Trace elements<br>10g Dextrose<br>218.6g Sorbitol<br>16g Agar
**CYA**	35g Czapek-Dox Broth<br>5g Yeast Extract<br>16g Agar
**YES**	5g Yeast Extract<br>30g Dextrose<br>16g Agar
**PDA**	24 g Potato Dextrose Extract<br>16g Agar

[a] 20x Nitrate Salts (1 Liter)

$NaNO_3$	(120g)
KCl	(10.4g)
$MgSO_4 . 7H_2O$	(10.4g)
KH_2PO_4	(30.4g)

[b] Trace Elements (100ml)

$ZnSO_4 .7H_2O$	(2.2g)
H_3BO_3	(1.1g)
$MnCl_2 . 4H_2O$	(0.5g)
$FeSO_4 . 7H_2O$	(0.5g)
$CoCl_2 . 5H_2O$	(0.16g)
$CuSO_4 . 5H_2O$	(0.16g)
$(NH_4)6Mo_7O_{24} . 4H_2O$	(0.11g)
Na_4EDTA	(5g)
