# Supplementary material for: New Insight Into Pathogenicity and Secondary Metabolism of the Plant Pathogen Penicillium expansum Through Deletion of the Epigenetic Reader SntB
Source: Front Microbiol. 2020 Apr 9;11:610. doi: 10.3389/fmicb.2020.00610 (PMC7160234; doi:10.3389/fmicb.2020.00610)
Supplement: Supplementary file 3 [file Data_Sheet_3.PDF]

Table S3. Plasmids used in this study

Name	Features	Reference
pBC-phleo	Phleomycin resistance gene *ble*	Silar (1995)
ß-rec6	Hygromycin resistance gene *hph*	Hartmann et al. (2010)
pJT1	Hygromycin resistance gene *hph* between 5' and 3' flanks of *P. expansum ku70*	This study
pJT2	Hygromycin resistance gene *hph and P. expansum sntB* ORF between 5' and 3' flanks of *P. expansum ku70*	This study
