# Supplementary material for: New Insight Into Pathogenicity and Secondary Metabolism of the Plant Pathogen Penicillium expansum Through Deletion of the Epigenetic Reader SntB
Source: Front Microbiol. 2020 Apr 9;11:610. doi: 10.3389/fmicb.2020.00610 (PMC7160234; doi:10.3389/fmicb.2020.00610)
Supplement: Supplementary file 4 [file Data_Sheet_4.PDF]

Table S4. Oligonucleotides used for making the deletion cassettes, molecular cloning and real-time PCR

(A) Primers used for constructing deletion cassettes, molecular cloning and mutants'confirmation	
**Primer**	**Sequence (5'-3')**
Pe_KOKu70_5'F	CGTAATCATGGTCATAGCTGTTTCCTGTGTCCCACGAAGAGTTCTTCGTTCTCC
Pe_KOku70_5'R	GGTTGTGAAACAGGGAACCTTAGAG
Pe_KOKu70_HygF	CCAATCCTCTAAGGTTCCCTGTTTCACAACCGCATCACTCAGGTCCTATAGGTC
Pe_KOKu70_HygR	ATATGATCCCAAGCAAACTTCAATCAGCGTGCACTAGATGGACCATATTATGCTCAAC
Pe_KOku70_3'F	ACGCTGATTGAAGTTTGCTTGGGATC
Pe_KOKu70_3'R	GCTCACATGTTCTTTCCTGCGTTATCCCCTTAGCTGCTTGTACCATAGACAGGGC
Pe_KOKu70_NestedF	GGCAGTCGATTAGCTCCGGAATC
Pe_KOKu70_NestedR	GCTTTGTTCAGCCGCTCAAATATCC
Pe_Ku70orf_F	GATGAAGAGCTTGACGAGACTGTGAG
Pe_Ku70orf_R	CCTGCTGTACATCATCATCAGCATCTG
hph_F	AAGCTGCATCATCGAAATTGCCG
hph_R	GCAAGGAATCGGTCAATACACTAC
Pe_KOstnB_5'F	TTGTATTCTCCTAGTCCATAGACCC
Pe_KOstnB_5'R	CAGGCTCGACGTATTTCAGTGTCGAAAGATCTGAGTGAGTCCCTTAGTCATCCAC

phleo_F	GATCTTTCGACACTGAAATACGTCGAG
phleo_R	GGATTACCTCTAAACAAGTGTACCTG
Pe_KOsntB_3'F	GAATGCACAGGTACACTTGTTTAGAGGTAATCCCGATATCCCCACATGAGTGCTC
Pe_KOsntB_3'R	GCAAGACCGAATACTTCATCTCCG
Pe_KOsntB_NF	TATCCGACAATCGCGTCCAAGG
Pe_KOsntB_NR	GATATTGAAGTCTTCGTGTCCGTCG
Pe_sntBorf_F	GCCAGCACGCCAAATTCAACTC
Pe_sntBorf_R	CGAGCTCCTCAATCTCTGACACG
Pe_sntBcomp_F	GGTGGTCCCGGGCCTAGTCCATAGACCCTCGATATCC
Pe_sntBcomp_R	ACCACCCCCGGGATAAAGTGGCCAATCCTACTCCATGC
Pe_sntB_probe_F	CTCGGCGCCAAACTCAAAGTCAG
Pe_sntB_probe_R	CCCTGTTCGAATAGCTTGAGCTCC
YS_F	CTCTTTCTTCTGGAGGAGATCTTCG
YS_R	GTGGCTTGGTCAAATGCACGAGC
**(B) Primers used for quantitative Real-Time PCR analyses**	
F28S	GGAACGGGACGTCATAGAGG
R28S	AGAGCTGCATTCCCAAACAAC

PEXP_005520 F (citS)	TGAACACCGCAACCCATTT
PEXP_005520 R (citS)	GCCTCGGATGGGTCTGGTA
PEXP_005550 F (cntA)	TCATGCCCATCCCATTGTTA
PEXP_005550 R (ctnA)	CGCGTCTGACCCTCGATTAC
PEXP_005590 F (citC)	TCGCAGACCGAGTGTTCCA
PEXP_005590 R (citC)	GGCTCACTCGTCGACCAATT
PatK F	GACGCTGGGCTACTGGATTG
PatK R	TCGTGCGTGAGGCCAGTAT
PatL F	GCAGGAGATCCGTTTCAGACA
PatL R	CCACTGACCGACGGTTACAAC
PatN F	CGTTCGATGTCGCTAGCAAA
PatN R	GGCGATAATCACGTCAATTCG
F1 LaeA-RT	TGGTAGTGTCGCAAGTTGGC
R1 LaeA-RT	TCGACCTGCTCAAACCAGG
F1 CreA-RT	AGAACATGACCCGTTCGCAT
R1 CreA-RT	TGGGTGAGTTGGGACGAGAG

FpacC-active	ATGTATACTATGCCCTGAGCCAA
RpacC-active	TCGTTCAACGCGTCATATCC
Beta-tubulin F	CTCCAGCTCGAGCGTATGAAC
Beta-tubulin R	GGCTCCAAATCGACGAGAAC
