# Supplementary figures and images for: New Insight Into Pathogenicity and Secondary Metabolism of the Plant Pathogen Penicillium expansum Through Deletion of the Epigenetic Reader SntB
Source: Front Microbiol. 2020 Apr 9;11:610. doi: 10.3389/fmicb.2020.00610 (PMC7160234; doi:10.3389/fmicb.2020.00610)

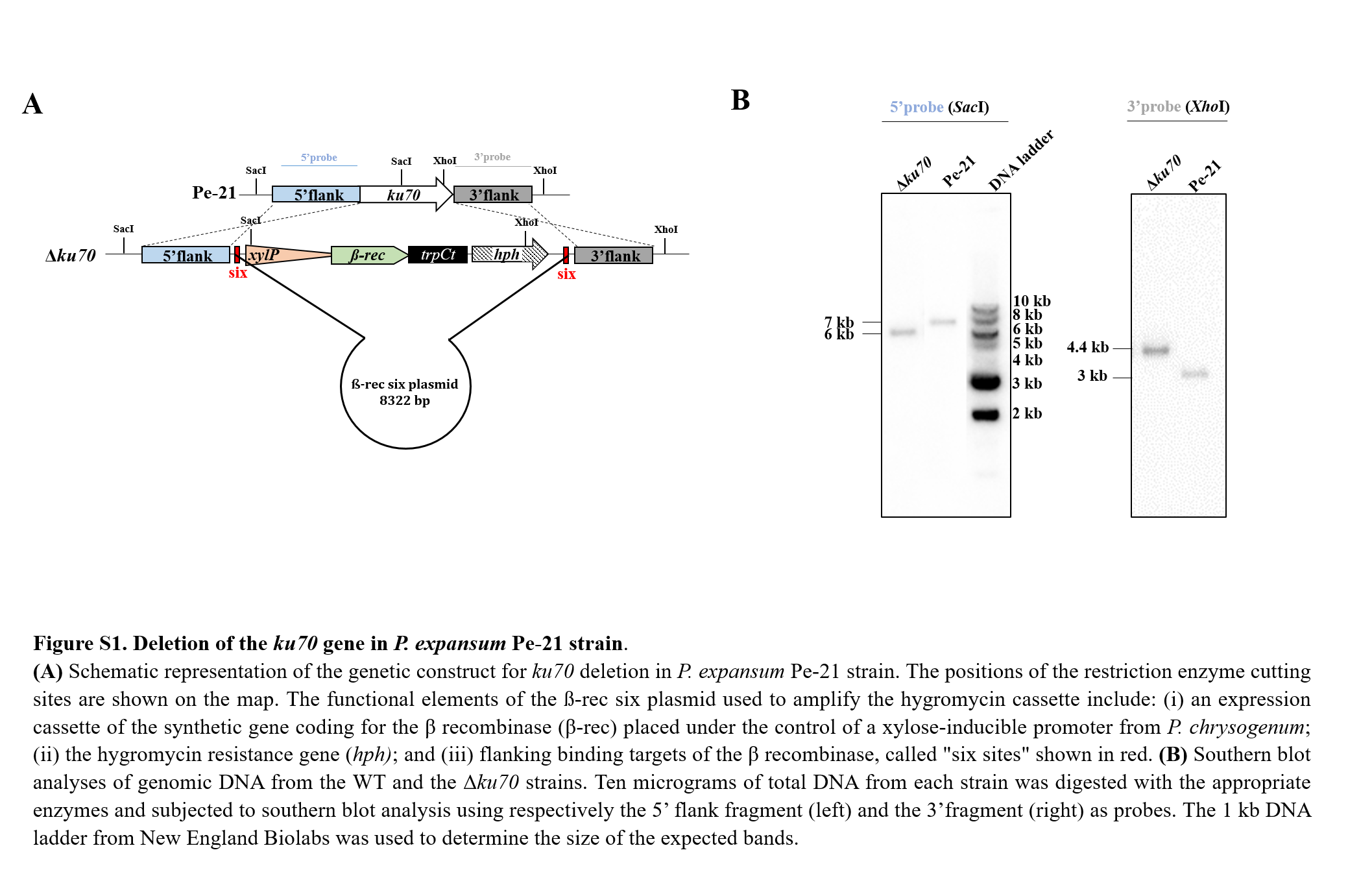

Supplement: Supplementary file 5 [file Image_1.TIF]

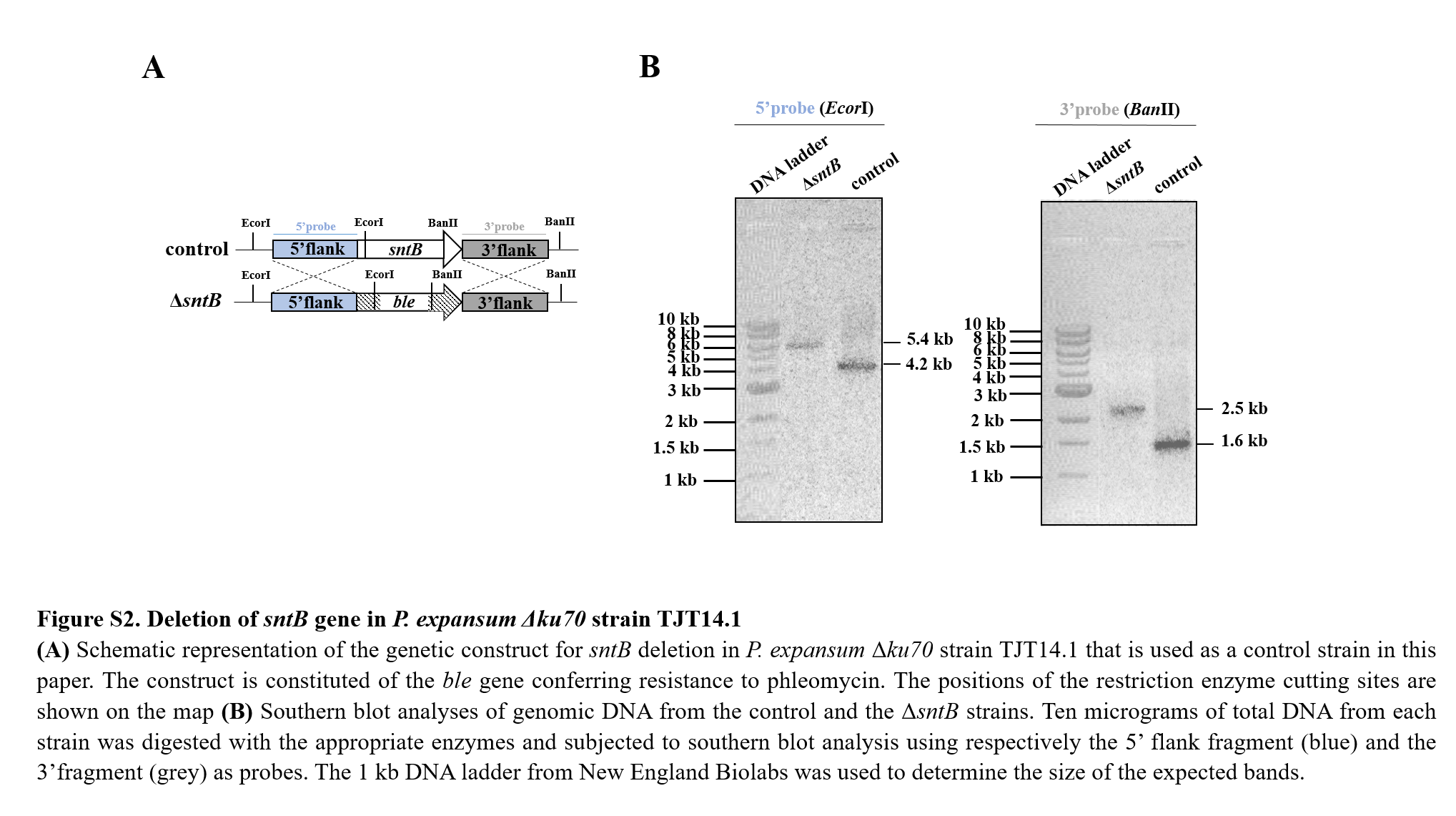

Supplement: Supplementary file 6 [file Image_2.TIF]

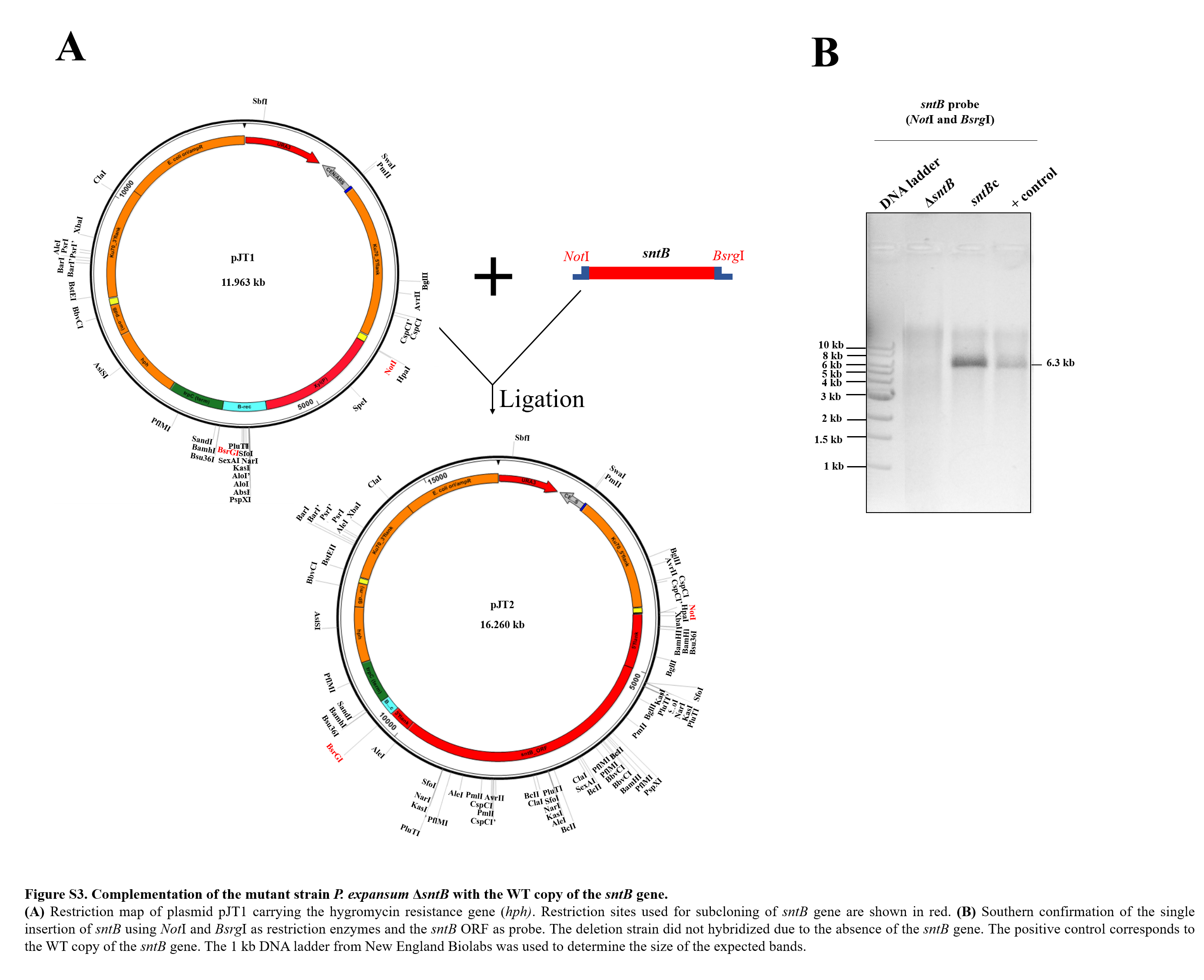

Supplement: Supplementary file 7 [file Image_3.TIF]

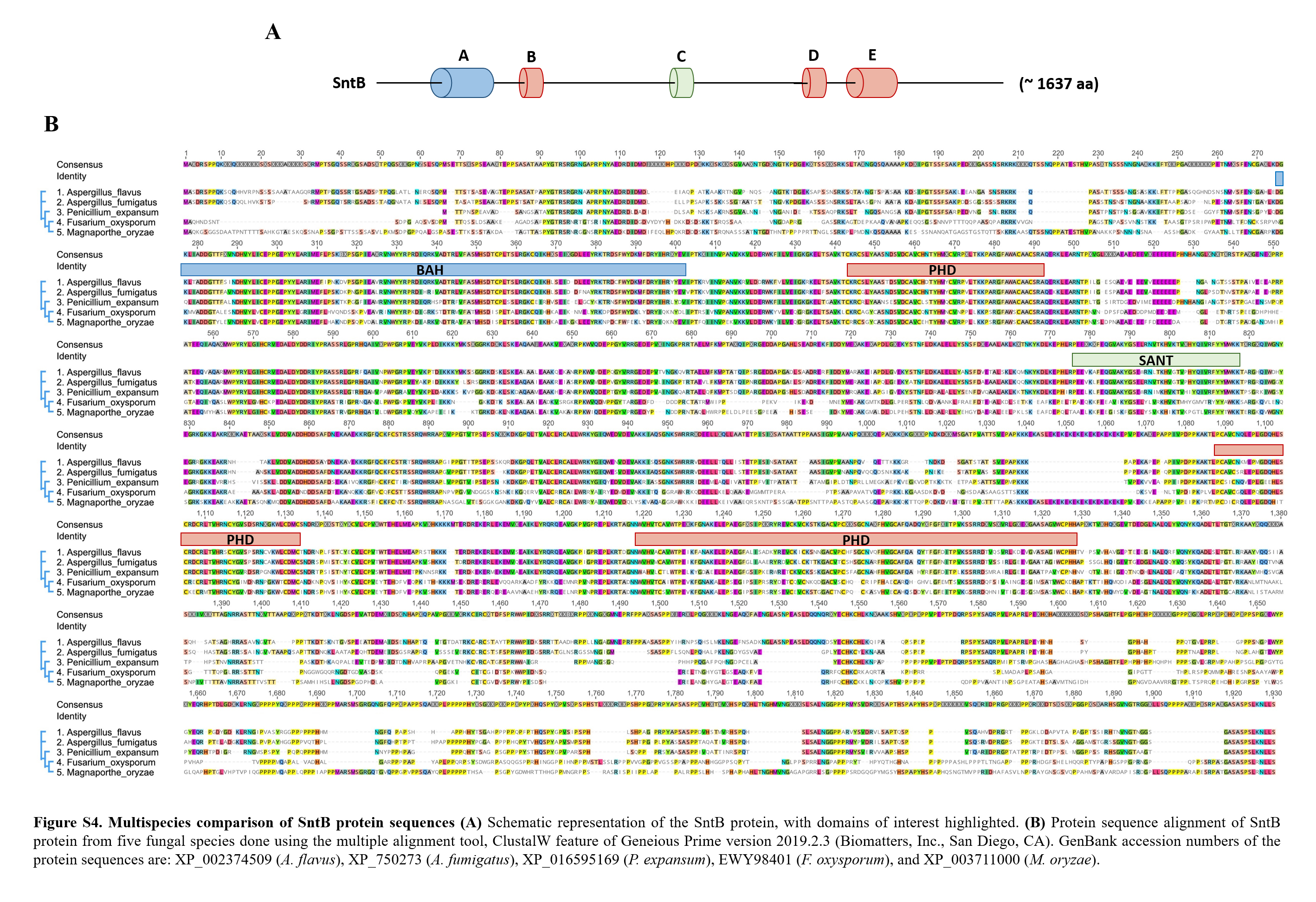

Supplement: Supplementary file 8 [file Image_4.TIF]

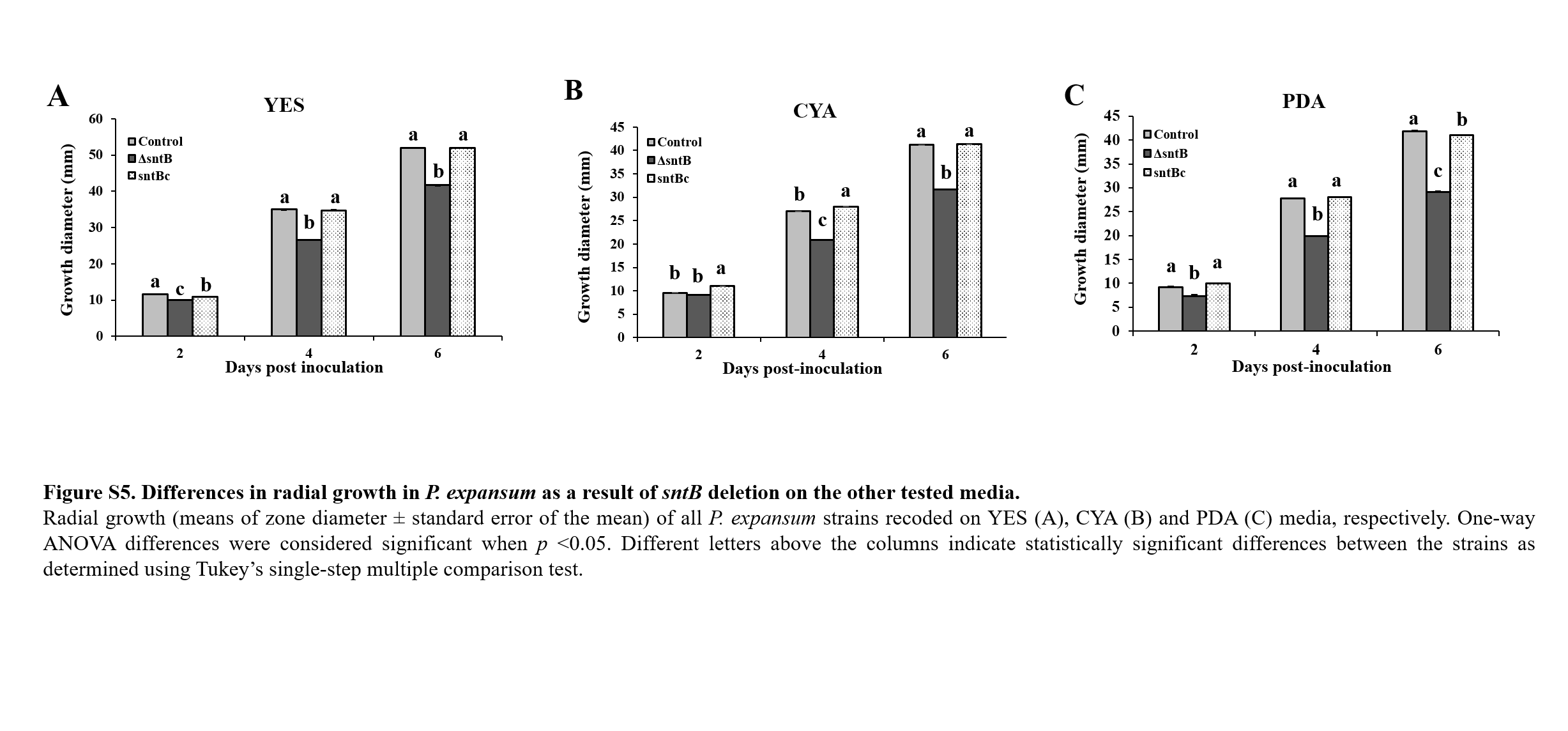

Supplement: Supplementary file 9 [file Image_5.TIF]

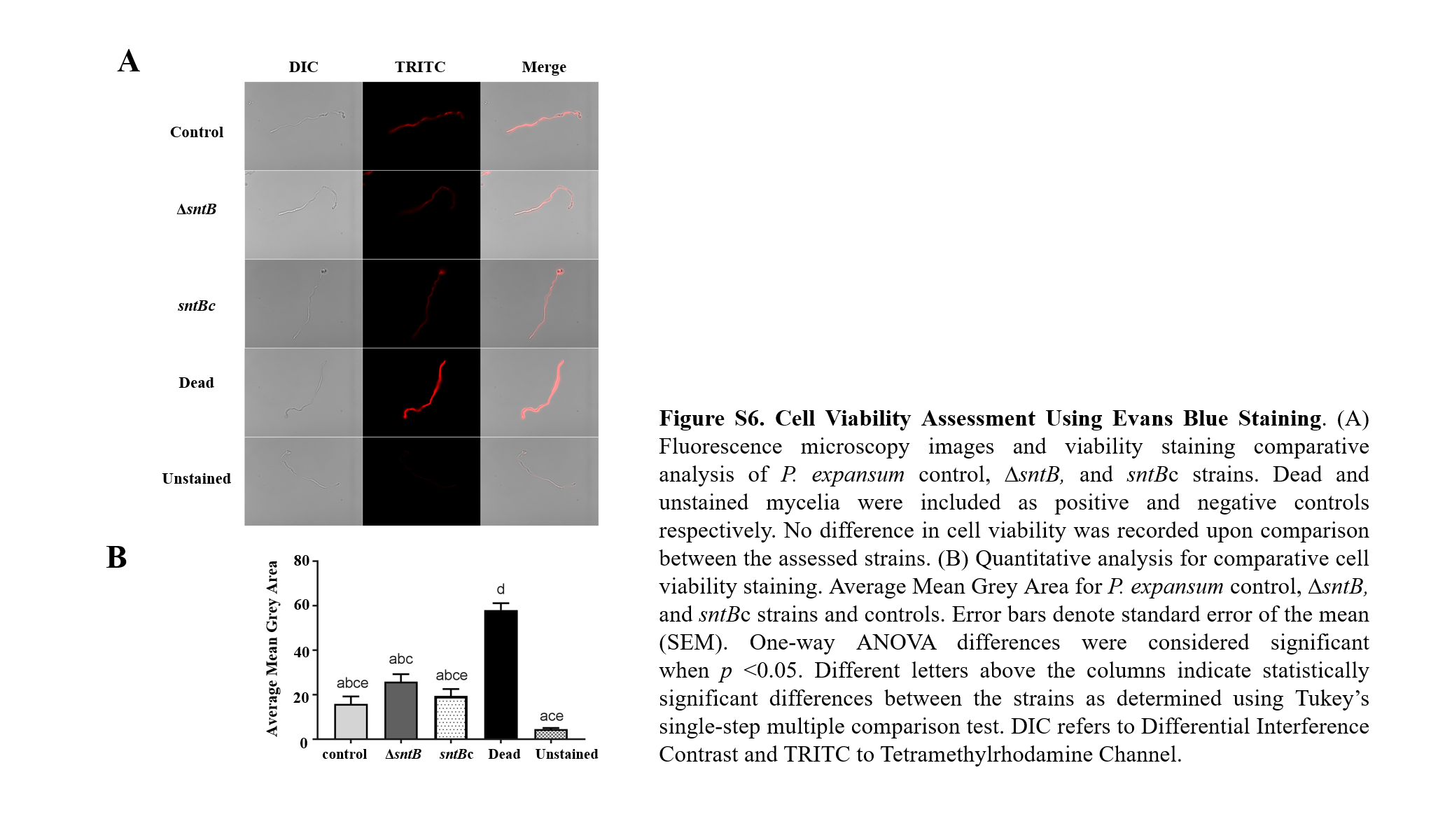

Supplement: Supplementary file 10 [file Image_6.TIF]
